# Label-free quantitative evaluation of breast tissue using Spatial Light Interference Microscopy (SLIM)

**DOI:** 10.1038/s41598-018-25261-7

**Published:** 2018-05-02

**Authors:** Hassaan Majeed, Tan Huu Nguyen, Mikhail Eugene Kandel, Andre Kajdacsy-Balla, Gabriel Popescu

**Affiliations:** 1 Quantitative Light Imaging (QLI) Lab, Beckman Institute of Advanced Science and Technology, University of Illinois at Urbana Champaign, 405 N Matthews, Urbana, IL 61801 USA; 2 Department of Pathology, University of Illinois at Chicago, 840 South Wood Street, Suite 130 CSN, Chicago, IL 60612 USA

## Abstract

Breast cancer is the most common type of cancer among women worldwide. The standard histopathology of breast tissue, the primary means of disease diagnosis, involves manual microscopic examination of stained tissue by a pathologist. Because this method relies on *qualitative* information, it can result in inter-observer variation. Furthermore, for difficult cases the pathologist often needs additional markers of malignancy to help in making a diagnosis, a need that can potentially be met by novel microscopy methods. We present a *quantitative* method for label-free breast tissue evaluation using Spatial Light Interference Microscopy (SLIM). By extracting tissue markers of malignancy based on the nanostructure revealed by the optical path-length, our method provides an objective, label-free and potentially automatable method for breast histopathology. We demonstrated our method by imaging a tissue microarray consisting of 68 different subjects −34 with malignant and 34 with benign tissues. Three-fold cross validation results showed a sensitivity of 94% and specificity of 85% for detecting cancer. Our disease signatures represent intrinsic physical attributes of the sample, independent of staining quality, facilitating classification through machine learning packages since our images do not vary from scan to scan or instrument to instrument.

## Introduction

The latest World Health Organization (WHO) figures have reported breast cancer as the second most common form of cancer worldwide with 522,000 deaths in 2012^1^. Within the US over 200,000 new cases of the disease are expected for women in 2017 according to the American Cancer Society^2^. Effective treatment strategies require timely and accurate diagnosis of the disease. It has been reported that, in the US, the 5-year average survival rates for patients with invasive breast cancers increase from 90% to 99% when the disease is detected at a localized (non-metastatic) stage^3^.

The standard tissue evaluation method for diagnosing breast cancers involves microscopic examination of a hematoxylin and eosin (H&E) counter-stained tissue biopsy. The biopsy specimen is obtained from the patient when suspicion of disease is noted during a screening procedure such as X-ray mammography. Since cells and histological tissue sections are transparent, the H&E stain provides the necessary contrast for assessing tissue morphology using a conventional bright field microscope. This standard histopathology process has two important short-comings: reliance on qualitative markers leads to intra- and inter-observer variation while manual examination can lower the throughput of the evaluation. Furthermore, evaluation of traditional H&E based markers is sometimes not sufficient for stratifying difficult cases and additional markers are needed to provide further information^4,5^. Novel quantitative microscopy methods can potentially help pathologists by offering an objective assessment of the tissue physical properties as well as new image markers for assessing difficult cases. Furthermore, quantitative markers can be interpreted by machine learning classifiers for rapid analysis and automated detection^6^.

In this work, we present a method for extracting quantitative markers of malignancy in breast tissue biopsies using Spatial Light Interference Microscopy (SLIM)^7^. SLIM is a quantitative phase imaging (QPI)^8^ modality that generates contrast by measuring the variation of optical path-length difference (OPD) across the tissue specimen. OPD reports on the product of the refractive index and thickness of tissue at each pixel. Malignant transformation involves physical changes in epithelial cell size and density as well as the tissue organization – both of which affect OPD maps of tissue. Therefore, these maps have been used in the past for several clinical investigations^9^, including diagnosis of prostate^10^ and colorectal cancers^11^, prediction of recurrence in prostate cancer^12^, analysis of Gleason grade^13^, assessment of metastatic pancreatic cells^14^, as well as detection of pre-malignancy in colorectal tissue^15^. Furthermore, using QPI, other live human cells have also been investigated for morphological^16,17^, chemical^17–19^ and mechanical markers of disease^20–22^.

To date, the majority of quantitative image analyses on breast tissue biopsies have relied on color images of stained tissue. Image classification in these cases has involved computing a wide range of histological features including geometric features^23,24^, texture-related features^25,26^ and radiometric features^25,27,28^ [see^29^ for a review of methods]. However, the feature extraction process relies heavily on tissue staining which can vary from sample to sample and instrument to instrument, affecting the robustness of the classifier^30^. The label-free approach we propose makes classification through machine learning easier since the instrument does not require calibration for inconsistency in pixel values due to variations in staining, tissue changes caused by harsh solvents etc. These advantages of QPI have already been leveraged to develop supervised learning methods for classifying erythrocytes infected with *Plasmodium falciparum*^31^ and non-activated lymphocytes^32^. Other label-free quantitative methods for tissue image classification have been proposed in the literature, including Fourier transform infrared spectroscopy (FTIR)^33–35^, Raman spectroscopy^36–38^, optical coherence tomography (OCT)^39,40^, and second-harmonic generation (SHG) imaging^41,42^. However, these techniques differ from our QPI-based method in terms of speed, resolution, and compatibility with the current diagnostic pipeline.

In our previous work we demonstrated that SLIM captures sufficient tissue morphology to separate benign from malignant tissue via visual investigation by trained pathologists^43^. In that work, two board certified pathologists diagnosed tissue microarray (TMA) cores as either benign or malignant by examining tissue morphology in SLIM phase images. 88% and 87% accuracy of diagnosis using SLIM was obtained for the pathologists, respectively^43^. In this work, we demonstrate the quantitative analysis capabilities of our tissue evaluation system by imaging a TMA comprising 68 different cases (34 benign and 34 malignant). For each epithelial region within a tissue core, we extracted scattering, geometric, and texture-related markers of tissue malignancy from the SLIM maps (see Materials and Methods). A linear-discriminant analysis (LDA) classifier was trained to separate benign cases from malignant cases and three-fold cross validation was performed to measure the classification accuracy of the learned model^44,45^. Using validation by the Receiver Operating Characteristic (ROC) curve analysis, our results revealed a sensitivity of 94% and specificity of 85%. Our results are the first demonstration, to our knowledge, of using OPD based tissue markers for detecting malignancy in breast tissue, label-free.

## Materials and Methods

### SLIM Optical Setup

**Figure 1:**
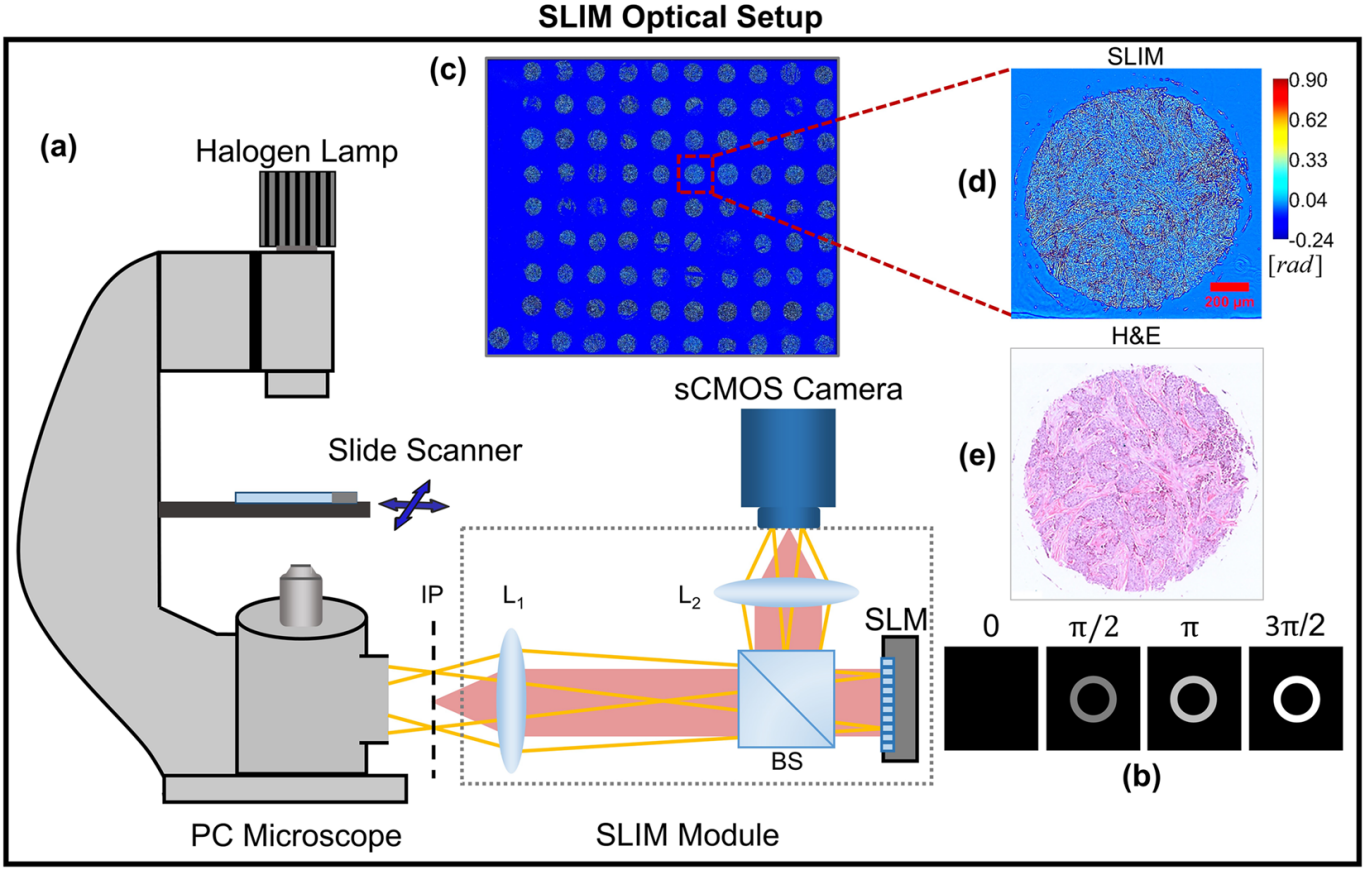
(**a**) The SLIM module added on to a commercial phase contrast microscope. (**b**) Four frames are acquired to compute one phase image by modulating the phase difference between scattered and incident light using a spatial light modulator (SLM). **(c)** An image of the whole slide scanned using SLIM. (**d**) Example of a TMA core SLIM image. (**e**) Bright field image of the same core after H&E staining. BS, beam splitter; L_1_-L_2,_ lenses; IP, image plane.

Figure 1 illustrates the SLIM optical setup which has been discussed in detail in previous publications^7,46^. The setup comprises a module (CellVista SLIM Pro, Phi Optics, Inc.) coupled to the output port of a commercial phase contrast microscope (Carl Zeiss, Axio Observer Z1). This compatibility with existing microscopes promises to reduce barriers to clinical adoption since optical microscopes are commonly available in pathology labs. In the SLIM module, the conjugate image plane outside the microscope is relayed onto a sCMOS camera (Andor, Zyla) using a 4f system comprising lenses L_1_ and L_2_. At the Fourier plane of L_1_, a spatial light modulator (Boulder Nonlinear Systems) is used to modulate the phase difference between the scattered and unscattered components of light in increments of π/2. Four different modulations are applied [Fig. 1(b)] and the resulting phase image is reconstructed using a previously published algorithm^7^. Using a software platform developed in-house, the SLIM module has been upgraded with full-slide scanning capabilities^11,43^. The acquisition speed is in the range of the existing commercial tissue scanners, which, in turn, only perform bright field imaging^11,43^. Throughout our experiments, a 40x/0.75 NA phase contrast objective was used for imaging. At this sampling rate (6.2 pixels/$\mu m$), the typical time for imaging a single tissue core (1 mm^2^ area) was approximately 12 sec.

### Tissue microarray

The TMA used for our study was purchased from US Biomax Inc. (Serial # BR-1002) with diagnosis for each case provided by the manufacturer through examination by a board certified pathologist. The TMA was obtained with all human subject information de-identified. Neither the authors of this work nor their institutions were involved in the collection of tissue. The TMA consisted of cores 1 mm in diameter and a section thickness of 5 µm. Standard formalin fixation and paraffin embedding (FFPE) histological preparation was used for each tissue block before extraction of cores. A xylene based mounting medium was used during cover-slipping.

The TMA consisted of 36 cases of infiltrating ductal carcinoma (IDC), 36 cases of tumor adjacent normal tissue and 10 cases of normal breast tissue derived from autopsy procedures (one core per case). Three of the tumor adjacent normal cores were obtained from the IDC cohort. The TMA was designed to mix approximately equal numbers of histologically normal and histologically invasive carcinoma cases. The post-mortem interval for autopsy cases was less than 6 hours and after assembly the TMA was inspected for quality control and diagnosis by the manufacturer through review by a board certified pathologist. For final analysis we selected 34 cores diagnosed as malignant and 34 cores diagnosed as normal (within which 28 were tumor adjacent normal and 6 were normal). Each of these final cores were selected based on whether the core was intact and whether any epithelial tissue was present in the core (cores containing only stromal tissue were excluded).

A SLIM image of the whole TMA slide is illustrated in Fig. 1(c). Figure 1(d,e) show, respectively, the phase map and the H&E stained tissue bright field image (henceforth referred to as ‘H&E image’) of one core. For obtaining a mosaic of the TMA, we used a C++ based stitching code, developed in-house^11^. After staining the same tissue slide using standard protocols^47^, H&E images of the TMA were acquired using a bright-field microscope (Carl Zeiss, Axio Observer Z1) outfitted with a color camera (Carl Zeiss, Axiocam MRC). The H&E images were used for qualitative evaluation only, to assist with annotation of epithelial regions in tissue, discussed below.

### Annotation of epithelial regions in tissue images

Glands or continuous epithelial regions within each core were manually annotated using the region of interest (ROI) tool of ImageJ to allow feature extraction for each gland. A consistent criterion for annotation was used where groups of epithelial cells bounded by stroma on all sides were considered a single gland. Other tissue components within epithelium (such as lumen etc.) were considered part of the gland if bounded on all sides by epithelial cells. Glands from cores in the cancer cohort were labelled as malignant while those from cores in the normal cohort were labelled as benign.

### Extraction of geometric and scattering features

**Figure 2:**
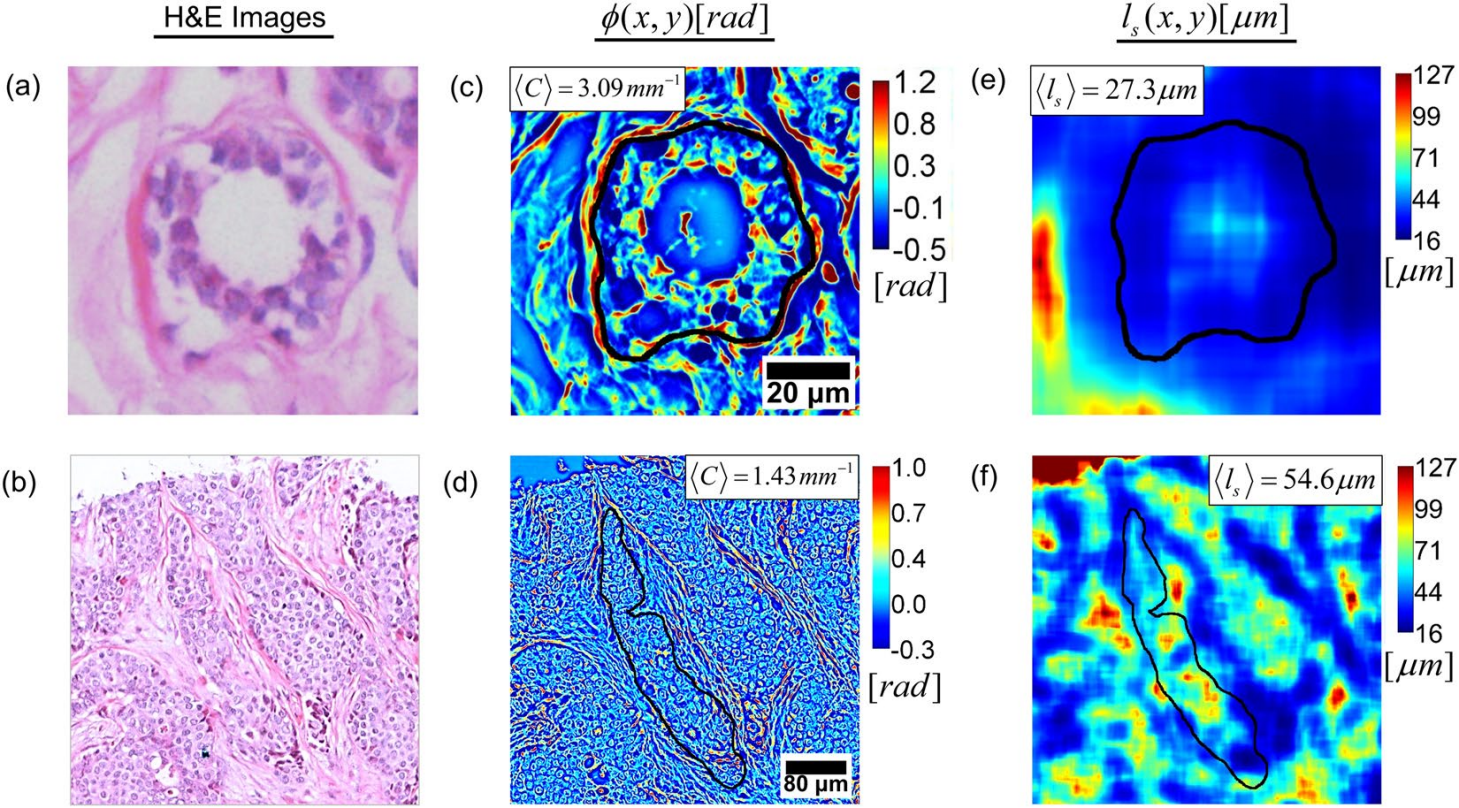
Computing the geometric feature $\langle C\rangle$ and scattering feature $\langle {l}_{s}\rangle$ over each annotated gland. (**a**) and (**b**) H&E images of benign and malignant glands, respectively. (**c**) and (**d**) SLIM images of the same benign and malignant glands, respectively, illustrating gland curvature $C$. The median over gland $\langle C\rangle$ is used as the geometric feature for classification. (**e**) and (**f**) ${l}_{s}(x,y)$ for benign and malignant glands, respectively. The median over gland $\langle {l}_{s}\rangle$ is used as the scattering feature for classification.

Malignant transformation in breast tissue affects the size, shape and density of epithelial cells as well as the shape and organization of epithelial tissue. As a result, both the geometry and scattering properties of the gland are affected. We used gland perimeter curvature $C$, as well as the mean scattering length ${l}_{s}$ as part of the feature set used for separating benign and malignant tissue. The parameter extraction process is illustrated in Fig. 2 and a detailed description for each is provided below.

The extrinsic curvature $C$ of a two-dimensional plane curve $P(x,y)$, that is parametrized by Cartesian coordinates $x(t)$ and $y(t)$ with parameter $t$, is given by the expression^48^

1 $$C(t)=\frac{|x\text{'}y\text{'}\text{'}-y\text{'}x\text{'}\text{'}|}{{(x{\text{'}}^{2}+y{\text{'}}^{2})}^{\frac{3}{2}}},$$

where the $x\text{'}$, $y\text{'}$ and $x\text{'}\text{'}$, $y\text{'}\text{'}$ refer to the first and second derivatives in $t$, respectively. In the above parametrization, $t$ refers to each pixel comprising the curve $P(x,y)$, having coordinates $x(t)$ and $y(t)$. This curvature can be interpreted as the magnitude of the rate of change of a vector tangent to $P(x,y)$. We computed $C$ for the perimeter $P(x,y)$ of each annotated gland by using an open source MATLAB code^49^. The code approximates $P(x,y)$ as a polygon before computing $C$ for each point defining the gland perimeter, as described in Eq. (1). To speed up computation, the image of each core was first down-sampled from the raw image size of 8000 × 8000 to 2048 × 2048 pixels. The sampling rate in the down-sampled image was 1.59 pixels/$\mu m$ and a bi-cubic interpolation technique was used for down-sampling. The perimeter $P(x,y)$ was then further down-sampled by a factor of 20 (every 20^th^ pixel was analyzed) before computing $C(t)$ in order to remove any pixel level errors due to manual annotation. The median gland curvature $\langle C\rangle$ was then used as a feature for separating benign and malignant cases. Figure 2(c) and (d) illustrate $\langle C\rangle$ for representative benign and malignant glands.

The scattering mean free path ${l}_{s}$, is a bulk scattering parameter that defines the length scale over which a single scattering event occurs on average. Assuming that the tissue slice captures the refractive index spatial fluctuation statistics, i.e., assuming statistical homogeneity, ${l}_{s}$ can be computed through the *scattering-phase theorem* using the expression^50^

2 $${l}_{s}=\frac{L}{\mathrm{var}[\varphi (x,y)]},$$

where $\varphi (x,y)$ is the SLIM phase image, $L$ is the tissue section thickness and the operator $\mathrm{var}[.]$ computes the spatial variance over a region. The ${l}_{s}$ parameter has been used in the past for discriminating between benign and malignant prostate tissue^10^. We first computed the image ${l}_{s}(x,y)$ from the phase image $\varphi (x,y)$ (8000 × 8000 pixels) using a variance filter kernel size of 149 × 149 pixels, which equals the approximate diameter of 3 epithelial cells. The feature $\langle {l}_{s}\rangle$ was then computed by calculating the median of ${l}_{s}(x,y)$ over the gland area. This computation is illustrated in Fig. 2(e,f).

### Extraction of texture-related features

**Figure 3:**
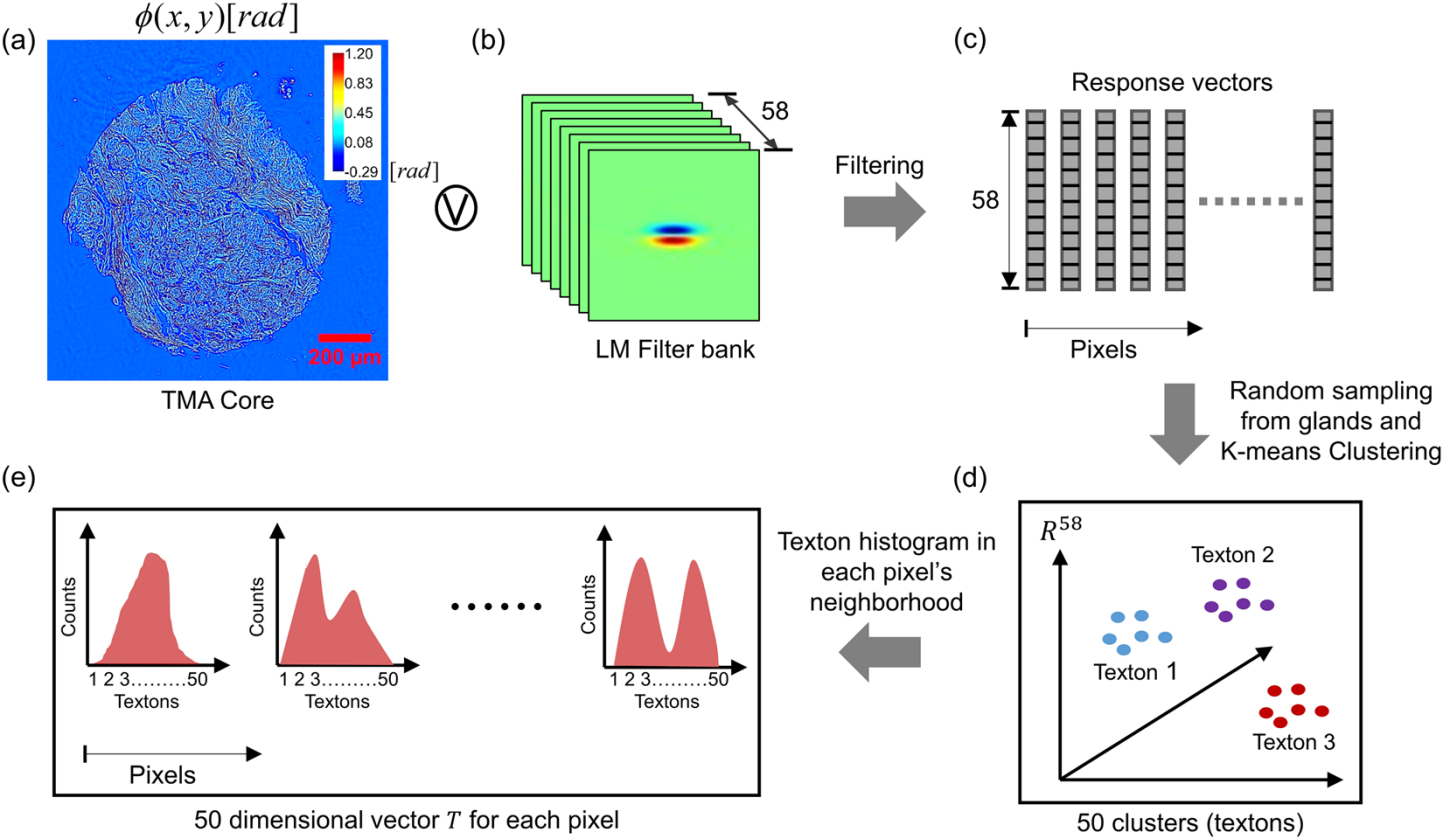
Algorithm for computing the texture in a pixel’s neighborhood. (**a**–**c**) Generating the response of each pixel to a Leung-Malik filter bank. (**d**) K-means clustering of response vectors, generated from all cores in the training set, in order to find 50 cluster centroids or textons. (**e**) Histogram of textons, within a pixel’s neighborhood, comprise the texture-related feature vector $T$ for each pixel.

Benign and malignant epithelial tissues differ not only in cell morphology but also in the organization of their components, leading to different textures. Texture-related features have been used in the past for solving different classification problems in histopathology of cancers^13,29^. Our feature extraction follows the work done by Varma *et al*.^51^ for classifying different materials based on their texture. The approach is illustrated in Fig. 3. Each TMA core phase image was first down sampled to 2048 × 2048 pixels from 8000 × 8000 pixels [Fig. 3(a)]. A bi-cubic interpolation was used for down-sampling and the sampling rate in the down-sampled image was 1.59 pixels/$\mu m$. The down-sampled core image was then filtered through a convolution with the Leung-Malik (LM) filter bank [Fig. 3(b)]. This filter bank consists of gradient filters (both odd and even) at different orientations and spatial scales^52^. In total, 58 different filters were used, generating a 58-dimensional response vector for each pixel in the core phase image [Fig. 3(c)]. The response vectors from epithelial regions within each core were then randomly sampled (10000 vectors per core) to generate a smaller dataset for further processing. K-means clustering was then performed on the response vectors (number of clusters, K = 50) sampled from all cores within each training set (see Results and Discussion) and the computed cluster centroids were referred to as ‘textons’^51,52^. K = 50 was chosen iteratively by repeatedly measuring the cross-validation AUC (see Results and Discussion) and determining the number of clusters required to maximize it (to account for both overfitting and separation accuracy). Since each pixel in each core belongs to a texton, for each pixel the histogram of textons was generated for its vicinity (window size 60 × 60 pixels) and was used to characterize the local texture in that neighborhood. This way, a 50 dimensional feature vector $T$ was generated to characterize texture in a pixel’s neighborhood. An open source MATLAB code was used for generating the LM filter bank for this work^53^.

### Classifier training and validation

**Figure 4:**
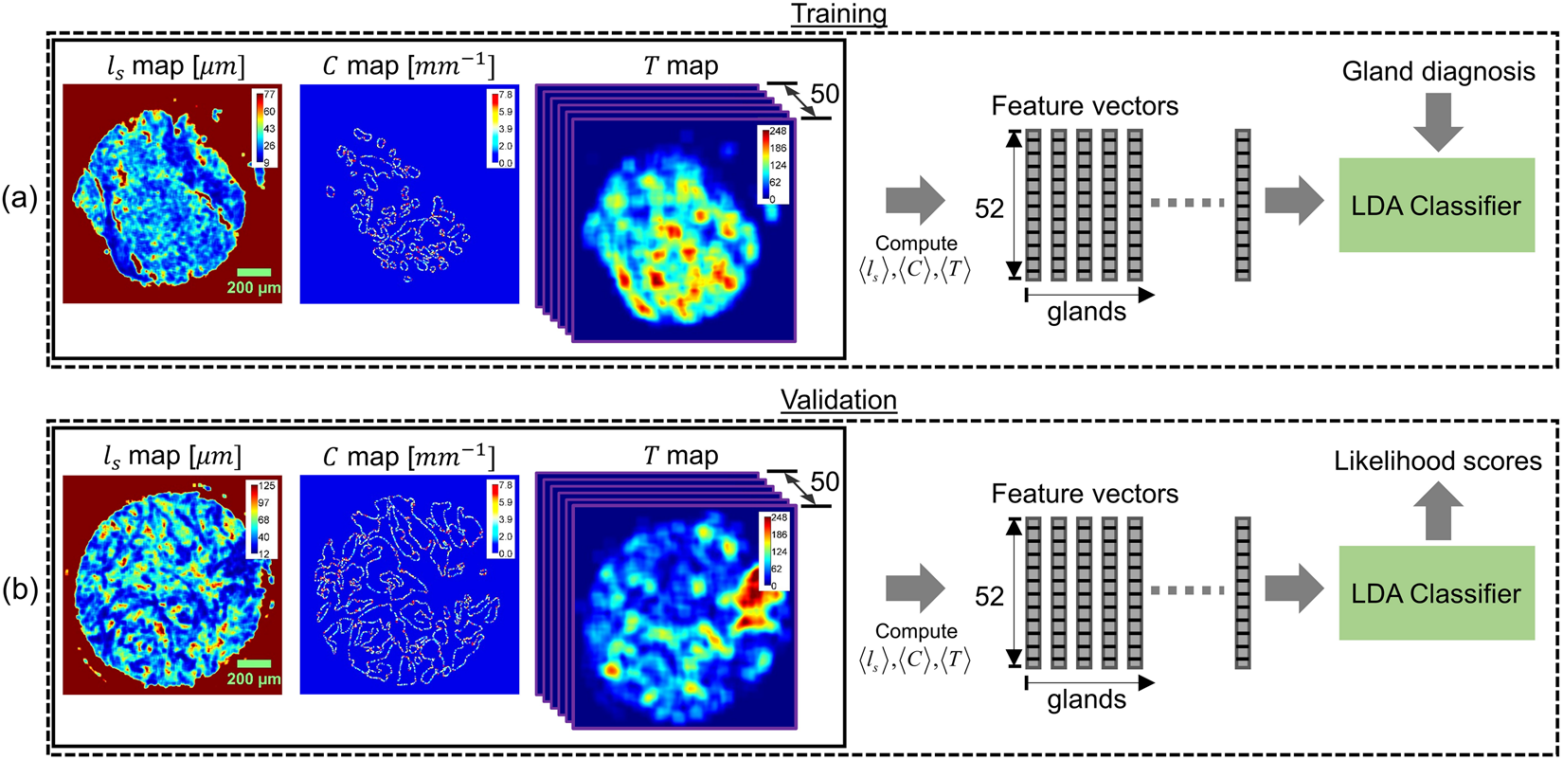
(**a**) Training and (**b**) Validation procedure for classifying glands as benign or malignant.

Since our work involves classifying each gland within a tissue core as benign or malignant, a feature vector for each gland was next generated by concatenating geometric, scattering and texture-related features. This procedure is illustrated in Fig. 4. After pixel-wise computation of gland curvature $C$, scattering length ${l}_{s}$ and texture vector $T$, the median of each feature was computed over each gland in a core and a combined 52 dimension feature vector was generated for training. For each gland, this feature vector was then used as a predictor for training a linear-discriminant analysis (LDA) classifier [Fig. 4(a)]. Class labels, either benign or malignant, were used as the ground-truth for each gland during the training process. All glands within cores deemed cancerous by the pathologist were labelled malignant and all glands within cores deemed normal were labelled benign.

The feature extraction for validation purposes, illustrated in Fig. 4(b), followed a nearly identical procedure to that used during training. The only difference was that, instead of finding new textons (cluster centroids) for validation data, the texture feature vector $T$ was computed by using the same textons as determined during training. As in training, a 52 dimensional feature vector was input to the LDA classifier which then used the model learned during training to generate a likelihood score for a gland being benign or malignant. Finally, the mean of the likelihood scores of all glands within a core was computed and used as the likelihood score of a core being benign or malignant. These scores were then used to generate a receiver operative characteristic (ROC) to select an operating point for separating benign and malignant cases (see Results and Discussion). For an annotated test core the total time required by our algorithm to generate a core likelihood score was approximately 2 minutes. This is in the absence of any parallelization of the computation through graphics processing unit (GPU) implementation which can significantly boost the computational throughput.

### Data availability statement

The datasets generated or analyzed during this work are available upon reasonable request.

## Results and Discussion

**Figure 5:**
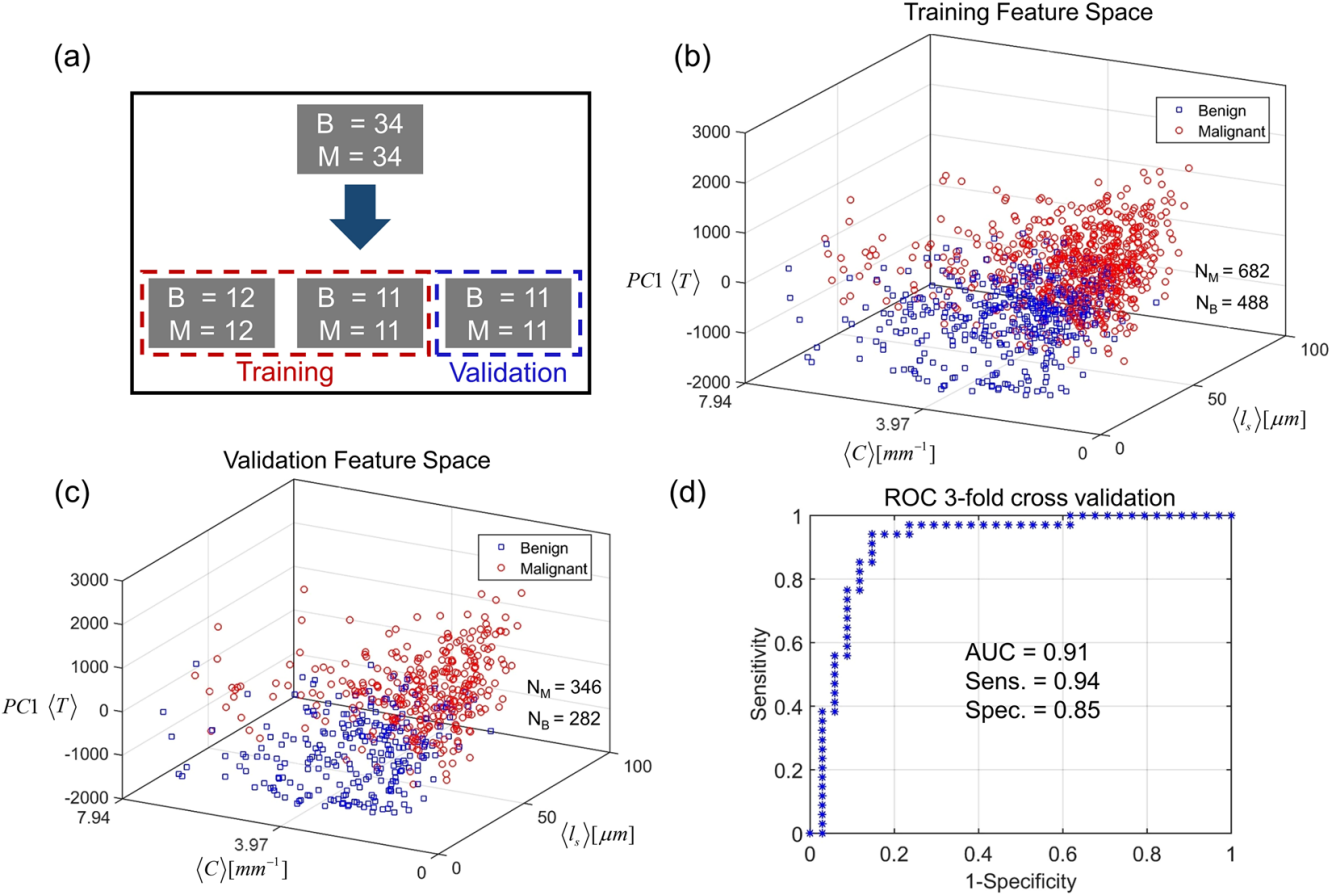
**(a)** Three-fold cross-validation procedure for evaluating classification accuracy. **(b)** Separation of benign and malignant gland feature vectors during *training* in 1 of 3 validation trials. **(c)** Separation of benign and malignant gland feature vectors during *validation* in 1 of 3 validation trials. **(d)** ROC curve for the 3 validation trials resulting in a sensitivity of 0.94 and specificity of 0.85 at the optimum operating point.

The classification results of our analysis are summarized in Fig. 5. In order to evaluate the accuracy of our method, we performed three-fold cross-validation^54^ as illustrated in Fig. 5(a). The total number of cases were divided into three (nearly) equal groups. In each trial, two groups were used for training while the remaining one was used for validation. Thus, three validation trials were performed, each time selecting a different validation/training set combination.

Figure 5(b) illustrates the separation between benign and malignant gland feature vectors in one training set. In order to illustrate the data separation in 3 dimensions, we use principal component analysis (PCA) and represent the 50-dimensional feature vector $\langle T\rangle$ through its first principal component PC1 $\langle T\rangle$. The training space shows that scattering feature $\langle {l}_{s}\rangle$ has on average higher values for malignant glands than for benign glands. This finding is compatible with typical gland morphology in breast tissue since benign glands are well differentiated, consisting of a number of different structures including epithelial cells, lumen and myoepithelial cells^55^. This heterogeneity of structure results in short mean scattering lengths as explained by a large variance in Eq. (2). Malignant glands on the other hand consist of a monoclonal proliferation of cells, sometimes even showing sheets of poorly differentiated epithelial cells, resulting in smaller variance and larger ${l}_{s}$ values^55^. These phenomena can also be observed in the examples given in Fig. 2(e,f). In previous investigations on prostate cancer, it was shown that ${l}_{s}$ has a lower value in malignant tissue than in benign tissue^10^. That analysis, however, was carried out on larger areas of tissue where cellular organization can be different from the epithelial only regions we are studying in this work^10^.

The median gland curvature $\langle C\rangle$, on the other hand, generally has higher values for benign glands than for malignant glands. This is a result of the fact that the edge of a benign gland is constrained to follow a round or elliptical shape due to tubule formation [Fig. 2(a) and (c)]^55^. When malignant transformation occurs, this constraint is broken and the gland edge is more irregular. At the spatial scale of investigation we have used here (approx. 13 $\mu m$), the perimeter of the malignant gland is less rapidly varying, on average, than that of a benign gland. This geometric feature is similar to the previous measurement of the gland perimeter fractal dimension that has been used for histopathology^23,29^.

Figure 5(c) shows the separation between benign and malignant glands in the validation feature space, where, qualitatively, the same separation trend is seen as in training. We show the results of only one of the three validation trials that were carried out. As described in Materials and Methods, the gland likelihood scores, generated by the classifier during validation, were averaged over each gland in order to obtain core-wise or case-wise scores. The standard deviation of gland likelihood scores, within each core, had a mean value of 0.18, a median value of 0.19 and a maximum value of 0.41 for the benign dataset (i.e across 34 cores). The same for the malignant dataset (again across 34 cores) were 0.17, 0.17 and 0.37, respectively. This indicates that the malignant cores consisted primarily (if not entirely) of malignant glands since the variance of gland likelihood scores over them was similar to that over benign cores, which consisted entirely of benign glands. In addition, while we treated all glands within cancerous cores as malignant during training, removing any benign tissue from malignant cores, during classifier training, is likely to improve our accuracy rather than worsen it.

The core-wise likelihood scores from the 3 trials were then pooled together to generate the ROC curve illustrated in Fig. 5(d)^56^. Our results indicate an area under the curve (AUC) of 0.91. The optimum operating point for classification was determined by using the standard method of assigning equal weight to the cost of misclassifying positives and the cost of misclassifying negatives^57^. This resulted in a sensitivity of 0.94 and specificity of 0.85 for the three-fold cross validation. When the same analysis was repeated by using the median rather than the mean of the gland scores over each core (to get a case-wise score), it resulted in an AUC of 0.91 and sensitivity and specificity of 0.94 and 0.82, respectively. While we use the standard method for determining the operating point here (which assigns equal cost to false negatives and false positives) in principle any operating point along the ROC curve can be chosen depending on the application (e.g 0.97 sensitivity and 0.77 specificity). Having said that, the higher sensitivity of 0.94 is useful since it results in a smaller number of false negatives than false positives. Minimizing false negatives is more important than minimizing false positives since the latter only result in further investigations of the patient whereas the former constitute a missed diagnosis. A sensitive method is also useful in situations where a small biopsy specimen is available and detection of small amounts of malignant tissue visually is a challenge for the pathologist.

Our results are significant because they are the first illustration of tissue OPD derived features being used to detect intrinsic markers of malignancy in breast tissue. While previous works employing image analysis and supervised learning for detecting cancerous regions in H&E-stained tissue images have demonstrated good classifications AUCs (greater than 0.90)^23,29,58,59^, accounting for stain variation through normalization remains a challenge^30^ due to a lack of universal agreement on the correct normalization method^29^. Our label-free results, thus, eliminate an important factor affecting consistency of results between different samples and instruments whilst maintaining high sensitivity and specificity.

## Summary and Conclusions

In summary, we presented a new method for quantitative evaluation of tissue biopsies obtained from patients under investigation for breast cancer. Since our method relies on measurement of OPD maps, an intrinsic property of tissue, the basis for classification is objective and not subject to inter-observer variation. In the past much of quantitative histopathology has relied on analysis of stained tissue. However, stain variability continues to remain a grand challenge in applying computer algorithms across multiple H&E stained specimens. This fact is well documented in the literature, as follows. In ref.^60^, it is stated that: “On the technical side, one of the main challenges in the computational interpretation of digital slide images has to do with color variations in the tissue induced by differences in slide preparation, staining, and even whole slide scanners. Clearly decision support algorithms that aim to work on digital pathology images will have to contend with and be resilient to these variations.” In ref.^61^ we find that “One of the major difficulties in breast cancer histopathology image analysis, particularly of H&E stained sections, is appearance variability.” As a final example, ref.^62^ states “It is clear that an integral part of digital pathology that has yet to be resolved is colour standardization; in order to do so, further work is needed focusing upon fine-tuning colour calibration methods in relation to the effect on diagnosis.”

Our method performs image processing and machine learning on unlabeled images, making it insensitive to variability due to staining. Although OPD depends on tissue slice thickness which can vary slightly from section to section, previous studies on colorectal and prostate cancer, using SLIM, have indicated that variations in OPD due to cutting errors are insignificant^11,63^. These studies looked at the variation of median phase values and anisotropy in scattering between tissue slices having the same nominal thickness and reported insignificant differences. Despite this preliminary evidence, future studies where variation in our feature set is explicitly tested against tissue slice thickness variation are required. While in this work we have performed manual segmentation of glands, the automation of the entire process (including segmentation) is feasible and subject to future efforts.

Our results demonstrate, for the first time to our knowledge, that QPI based markers can be used for detecting malignancy in label-free breast histology samples at high accuracy. While our cross-validation results show promising sensitivity and specificity, a number of further studies are proposed before clinical adoption of our method. First, our analysis needs to be further tested with separate training and testing tests, the latter being obtained from an independent laboratory and remaining unused during model development. Second, our feature set needs to be applied to more clinically difficult cases since making frank benign versus frank malignant diagnoses is not currently a serious challenge for pathologists. However, our results demonstrate an important and necessary first step and we propose to apply our feature set, in future studies, to more challenging cases such as stratification of benign lesions as well as distinguishing ductal carcinoma *in situ* (DCIS) from ductal hyperplasia atypia^4,5^. Third, while we have applied our diagnosis method to cases of IDC, the most widely prevalent form of breast cancer, the applicability of the model to other histological sub-types (such as infiltrating lobular carcinoma) also needs to be explored.

While other label-free diagnosis methods have been proposed for these types of investigations, they affect the standard diagnostic pipeline in terms of either speed, resolution or compatibility with established workflow. SLIM, on the other hand, requires minimal changes to a conventional microscopic optical train due to its modular design. Even though in this study a research grade microscope was coupled to the SLIM module, in principle SLIM can be used with any phase contrast microscope. Furthermore, the SLIM instrument is less expensive than most commercial tissue scanners. Our results, although preliminary, are an important stepping stone towards extracting reliable novel markers that can provide pathologists adjunct information to H&E based markers for assessing difficult cases. In addition, computational pathology tools such as ours can help pathologists where the biopsy specimen is small and highly sensitive detection is desired.
